# Finnish nursing students’ perceptions of the development needs in palliative care education and factors influencing learning in undergraduate nursing studies – a qualitative study

**DOI:** 10.1186/s12904-022-00915-6

**Published:** 2022-03-23

**Authors:** Minna Hökkä, Juho T. Lehto, Helvi Kyngäs, Tarja Pölkki

**Affiliations:** 1 Faculty of Medicine, Research Unit of Nursing Science and Health Management, University of Oulu, PO BOX 5000, 90014 Oulu, Finland; 2 Kajaani University of Applied Sciences, Kajaani, Finland; 3 Faculty of Medicine and Health Technology, Tampere University, Tampere, Finland; 4 Palliative Care Centre and Department of Oncology, Tampere University Hospital, Tampere, Finland; 5 Medical Research Center Oulu, Oulu University Hospital, Oulu, Finland

**Keywords:** Palliative care, Education, Nursing, Student

## Abstract

**Background:**

Nurses have an essential role in providing high-quality palliative care to patients and their families. Hence, they require adequate palliative care education. However, there is only limited insight into how final-year nursing students perceive palliative care education in undergraduate nursing programs. This study aimed to describe nursing students’ perspectives of the development needs of palliative care education. An additional two aims emerged based on the collected data, namely, to describe the preferred education for palliative care and the factors which promote or hinder palliative care learning during undergraduate nursing studies.

**Methods:**

The research was guided by a descriptive qualitative approach and applied inductive content analysis. The frequencies (f) of identified codes (reduced expressions) were counted to show the noteworthiness of each category in relation to the entirety. The participants were final-year nursing students (*n* = 766) who had participated in a national survey.

**Results:**

The inductive content analysis identified three unifying categories. The first was ‘Development needs and views of palliative care education’ (f = 524), which consisted of the main categories ‘the need to develop palliative care education’ (*f* = 414) and ‘meaning of palliative care and its education’ (*f* = 110). Secondly ‘Preferred types of palliative care education’ (*f* = 1379), including the main categories ‘teaching contents in palliative care education’ (*f* = 905), ‘teaching methods for palliative care learning’ (*f* = 393), and ‘placement of palliative care studies’ (*f* = 81). Thirdly ‘The facilitators and barriers to palliative care learning’ (*f* = 401), consisting of the main categories ‘factors facilitating palliative care learning’ (*f* = 66) and ‘barriers to palliative care learning’ (*f* = 335).

**Conclusions:**

This study provides detailed information about nursing student’s perspectives of palliative care education and its development needs. Hence, the results are relevant to decision-makers who want to develop undergraduate nursing curricula. This study highlights that palliative care education should be developed by ensuring that all students have equal access to palliative care education provided by highly competent teachers. Possibilities for clinical placements or visits to palliative care units during the education should also be improved. The participating students felt unprepared to provide high-quality palliative care even though they responded that palliative care is an important topic in their nursing studies.

**Supplementary Information:**

The online version contains supplementary material available at 10.1186/s12904-022-00915-6.

## Background

The need to develop palliative care education was highlighted by the Council of Europe (CoE) during an assembly in 2018. Access to palliative care should be a human right and, therefore, the provision of palliative care should be integrated into the health care system [1]. Furthermore, education is one of the core components in the World Health Organization’s public health strategy to successfully integrate palliative care into health care [2, 3] and has also been reported to facilitate the development of palliative care [4]. Several contemporary phenomena, i.e., an aging world population, the increase in noncommunicable diseases, and the emergence of novel viruses, recently highlighted by the COVID-19 pandemic, demonstrate that there is an immense demand for palliative care, which is expected to double by 2060 [5].

Nurses, as the largest occupational group in health care, have an essential role in ensuring high-quality care [6]*.* Nurses are also the primary providers of palliative care for patients and their families in many different contexts [6–8]. Hence, they should have sufficient palliative care education and competencies to provide high-quality palliative care [5, 9, 10]*.*

To ensure that all nurses possess the required palliative care competencies, undergraduate nursing education should cover palliative care [11, 12]*.* Nevertheless, previous research has reported that undergraduate nursing students lack competence to provide palliative care [13, 14] and feel unprepared for palliative care and encountering death [15–17]*.*

The need to develop palliative care education has previously been highlighted [18]. For example, the European Association for Palliative Care task force group published a report focusing specifically on the development of palliative care in nursing education [19]. Although this report is known in the European countries, there is still large variety in palliative care education both across and within countries. Notably, over half of European countries (56%) reported in a recent survey that palliative care was not a mandatory subject in undergraduate nursing education [20]. In Finland, universities of applied sciences (UASs) largely vary in terms of how palliative care is covered in undergraduate nursing programs*.* As a result, the recent statement of the Ministry of Social Affairs and Health includes recommendations for how Finnish nursing education should be developed [21]*.*

Previous evidence has shown that palliative care education increases nursing students’ knowledge of palliative care and positively influences their attitudes towards end-of-life care [18, 22–24]. Furthermore, palliative care education promotes students’ personal growth and self-awareness [25]. The need to integrate palliative care into undergraduate education has been emphasized before, with attempts at including this part of care into nursing curricula intensifying during the last 10 years [18]. Nevertheless, there is still an evident need to develop the extent to which palliative care is integrated into undergraduate nursing education [11, 18, 20, 21].

Most of the qualitative research on the topic of palliative care in nursing education published in the last 10 years has focused on students’ experiences of different teaching interventions, e.g., simulations or elective courses [26, 27], and students’ perspectives or attitudes towards end-of-life care or care for the dying patients [28, 29]*.* To our knowledge, there is only limited qualitative evidence of final year nursing students’ perspectives of the development needs in palliative care education in undergraduate nursing programs and how this area of care could be better integrated into the nursing curriculum.

## Methods

This study aimed to describe nursing students’ perspectives of the development needs of palliative care education. An additional two aims emerged based on the collected data, namely, to describe the preferred education for palliative care and the factors which promote or hinder palliative care learning during undergraduate nursing studies. The research applied a descriptive qualitative approach with an inductive content analysis method [30]. The intention of the study was to present a comprehensive summary of the phenomenon of interest without claiming any methodological roots [31].

### Settings

The study was performed across all of the Finnish UASs (*n* = 21) providing an undergraduate nursing program. In Finland, all registered nurses are required by law to have completed a Bachelor’s degree nursing program from a UAS [32]. After graduation the registered nurses need to apply for licensing to work as a nurse in Finland. The Bachelor’s degree program lasts approximately three-and-a-half years and comprises 210 European Credit Transfer and Accumulation System (ECTS) credits and fulfills the criteria of the European Union Directive 2013/55/EU [33]. The education includes 90 ECTS of clinical training. The education is free-of-charge and is funded by the Ministry of Culture and Education. The nursing teacher’s educational requirements are also regulated by law, e.g., they must hold a Master’s degree and have at least 3 years of working experience in the nursing field [34].

The UASs have autonomy in developing the undergraduate nursing curriculum. However, criteria set by the European Union [33] and a national consensus-based report of nursing competencies published in 2015 [35], and renewed in 2020 [36], serve as a framework for curriculum development. The renewed report of competencies includes palliative care, which was not so clearly addressed in the previous version. Registered nurses can deepen their competencies according to their own interests during their undergraduate nursing studies by choosing courses in the limits of the curriculum. Still, when graduating they all are registered nurses without specialization.

### Participant characteristics and data collection

The data were collected as part of a larger national cross-sectional survey that targeted undergraduate nursing students. The inclusion criteria for participation were that the student is in the final year of studies and is enrolled in nursing degree program in a Finnish UAS provided in either of the official languages in Finland (Finnish or Swedish). Detailed participant characteristics are shown in Table 1.

**Table 1:** Characteristics of the responding students

Number of respondents	766	
Age in years, median (range)	25	(20–58)
Gender, n (%)		
Female	678	(88.5)
Male	80	(10.4)
Did not define	8	(1.1)
Previous health or social care education, n (%)		
None	432	(56.4)
Practical nurse	307	(40.1)
Other education	27	(3.5)
Previous work experience in social or health care, n (%)		
None	273	(35.5)
Work experience	491	(64.3)
Unanswered	2	(0.2)

Data collection ran from September 2018 to March 2019. The participants consisted of a convenience sample of final year nursing students studying in a UAS at the time of data collection. Convenience sampling is a commonly used sampling method when recruiting participants from a particular setting. The sampling method aims to provide a group of participants with experience of the study phenomenon. In this study, the setting was UASs and the participants were final year nursing students, who had sufficient experience with nursing education to evaluate the development needs of palliative care education [31]*.*

Each UAS named a contact person who would perform the data collection. In 19 of the UASs, the contact person (first author in two UAS) distributed the paper questionnaire to final-year nursing students during a teaching session in a classroom, with the students answering the questionnaire during this session. In two UASs, students responded to the questionnaire online through a link sent via email to the student group by the contact person, with one follow-up message sent later to remind the students to complete the questionnaire. An information letter containing the study purpose, researchers’ contact information, including a description of their occupation, academic position, and interest in developing palliative care education, was attached to the survey. The students responded to the questionnaire completely anonymously, with no personal data being collected and no prior relationship was established between the researchers and any of the students.

The contact persons estimated that there were 1868 final-year students studying at the time when the questionnaire was provided to the student groups. A total of 1331 students gave an eligible response to the survey, of which 766 (57,6%) answered the open-ended, qualitative question included in this sub-study. The survey included six background questions, questions of students view of the palliative care contents provided in nursing studies and their self-assessed competence about palliative care, and an open-ended question: “Tell your thoughts on how palliative care education should be developed”. The data of this sub-study consisted of students written answers to this open-ended question. The length of the students written answers varied from one sentence to half of a Microsoft Word A4 page. The questions were developed by the authors. The clarity of the questionnaire was pretested by one student group (*n* = 15), no amendments were required based on the pretest. Information covering the characteristics and relevant experience of the authors can be found in Table 2.

**Table 2:** Researcher characteristics

Author (gender)	Credentials, Occupation at the time of the study	Education on qualitative research	Experience in qualitative research
(MH) (female)	RN (Master’s degree), MNSc, PhD-candidate Head of School at a University of Applied Science	Has completed formal qualitative research study modules at Master’s and PhD levels.	Has conducted qualitative research studies. Has taught qualitative research methods. Has supervised Bachelor’s and Master’s theses which have used qualitative research methods.
(JL) (male)	MD, PhD, Professor in a University and Chief Physician in a University Hospital	Has studied the principles and application of qualitative research methods through informal learning activities.	Has conducted qualitative research studies. Has experience in developing measurement tools and questionnaires.
(HK) (female)	RN, PhD, Professor in a University	Has completed formal qualitative research methods education at Master’s and PhD levels.	Has conducted qualitative research studies. Has taught qualitative research methods at Master’s and PhD levels. Has supervised Master’s and PhD theses which use qualitative research methods. Has authored a textbook and conducted research about content analysis as a research method. Has experience in developing measurement tools and questionnaires.
(TP) (female)	RN, PhD, Professor in a University	Has completed formal qualitative research methods education at Master’s and PhD levels.	Has conducted qualitative research studies. Has supervised Master’s and PhD theses which use qualitative research methods. Has experience in building measurement tools and questionnaires.

### Data analysis

The qualitative analysis was performed by an inductive content analysis method, in which categories emerge from the data and no theoretical framework is used as a starting point [30, 37]. The students’ responses (only manifest content) were analyzed to describe the phenomenon of interest. Words, sentences, or phrases which constructed a meaning were used as the unit of analysis [30]*.* No software was used in the analysis process.

In the first phase of analysis, the data were transcribed verbatim from the questionnaires to a Microsoft Word template, after which the researchers carefully read through the resulting data several times to become familiar with the content. During this phase of the analysis, the researchers noticed that the students’ responses reflected the development needs of palliative care education; moreover, they also provided their preferences for how the education should be delivered, along with factors that promoted or hindered their learning. In qualitative research it is possible that the aim and research question can change during the analysis process, i.e., the data direct the process [30]. Therefore, the aims of the present study were expanded to cover students’ preferences for how palliative care education should be provided and which factors promote or hinder their learning.

In the second phase of the analysis, the original data were reduced to codes, which were relevant to the study aims. In the third phase of the analysis, the reduced codes were grouped together based on similarities in content. The fourth phase of the analysis was data abstraction, i.e., sub-categories, categories, main categories and unifying categories were formed based on the grouped codes. The identified categories were inductively derived from the data, while the abstraction was performed in a way that it applied to all data [30, 37]*.* An example of the coding process is provided in Table 3.

**Table 3:** Example of the coding procedure, how the subcategory ‘Importance of genuine encountering’ was inductively produced

Example of the original data	Example of the resulting code (reduced expressions)	Subcategory in which the code was categorized
w46 being heard is really important in terms of a successful, genuine encounter.	w46 being heard is important for genuine encounters	
w61 A genuine encounter is important.	w61 Genuine encounters are important.	
w57 Usually it is enough that we are genuinely present for the other person.	w57 It is enough to be genuinely present.	Importance of genuine encounters *(f = 5)*
w57 Usually it is enough that we are genuinely our own selves for another.	w57 Importance of genuinely being yourself for others.	
1129 More emphasis should be placed on …that time should be provided for genuine encounters with the patient.	1129 *(more emphasis)* to arrange time for genuine encounters with patients	

f, number of codes included in the subcategory

The coding process was performed by one of the authors (blinded), after which all other members of the research group went through the results. Once consensus was achieved, the first author performed the categorization and abstraction of the data, which was again critically assessed by all members of the research group. The frequencies (f) of codes (reduced expressions) were counted to show the noteworthiness of each category in relation to the entirety. The collected data yielded a total of 2304 codes. Data saturation, which is the point at which no further data collection is necessary, was achieved during the analysis process [38]*.* The research adhered to COREQ guidelines (Additional file 1) for the reporting of qualitative research [39]*.*

### Ethical consideration

The standards of the Declaration of Helsinki were followed during each step of the study [40]*.* Each participating UAS (*n* = 21) granted a written study permission for the data collection. Before starting data collection, the Ethical Committee of North Ostrobothnia’s Hospital District was consulted regarding the need for a separate ethical statement. It was not needed since, according to Finnish law, a statement is not required when the study does not intervene with participants’ integrity [41]*.* The participants were informed about the voluntary nature of participation in the study and received written information about the study aims. Each student responded that they had read the information letter and agreed to participate in the study by answering ‘yes’ to a question of this issue. If the question was left unanswered or the answer was ‘no’, the response was rejected. The researchers’ contact information was included in the information letter in case of questions or concerns of the students related to the study. Since data collection was anonymous, no personnel register was formed in the study and anonymity was protected so that the student cannot be identified through the examples of authentic data presented in the study [42].

## Results

**Fig. 1:**
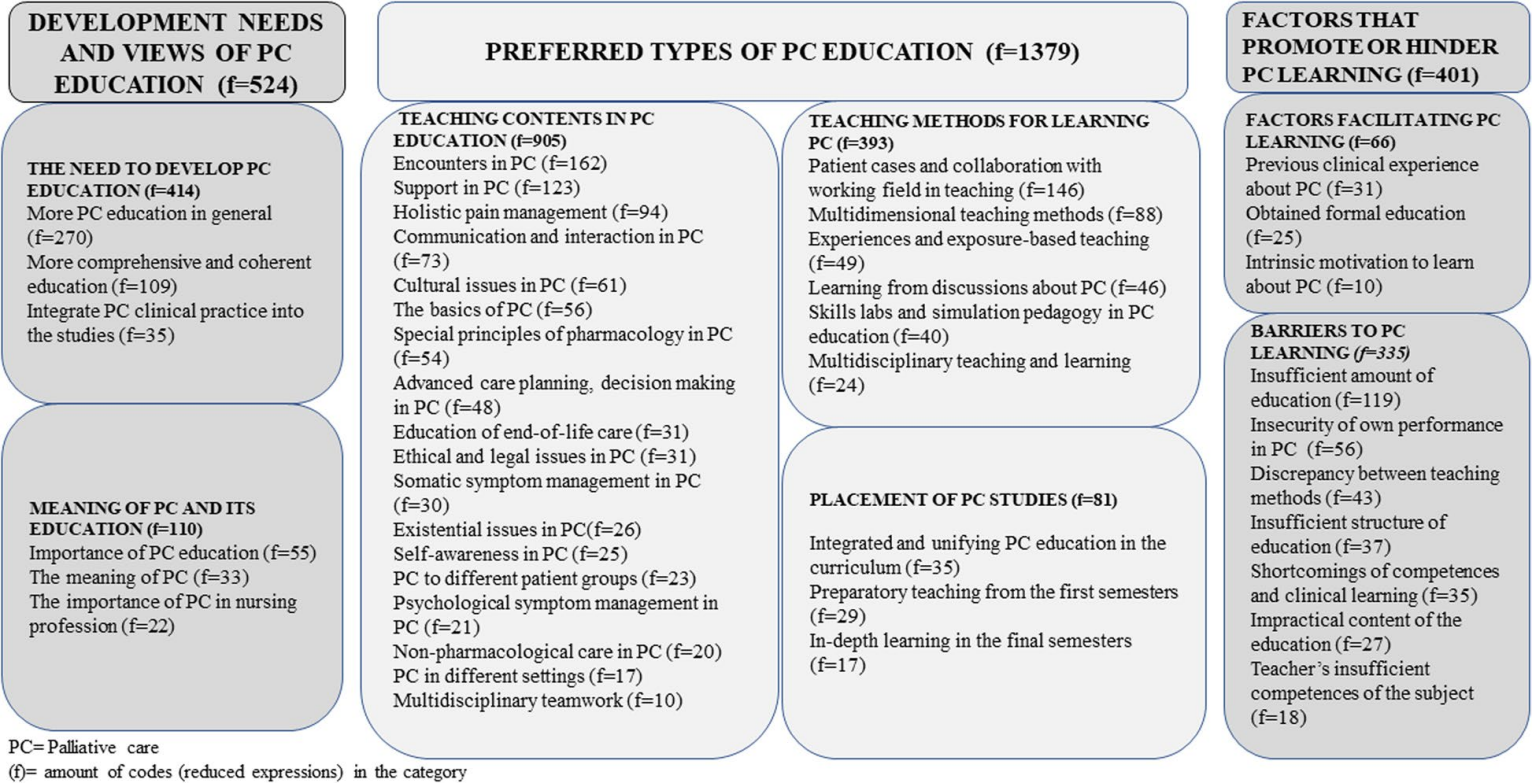
Students perspectives of palliative care education and its development needs

Three unifying categories were identified, namely, 1) development needs and views of palliative care education (*f* = 524), 2) the preferred types of palliative care education (*f* = 1379), and 3) factors that promote or hinder palliative care learning (*f* = 401) (Fig. 1). Each subcategory presented below will include one quotation as an example.

### Development needs and views of palliative care education

This unifying category included two main categories: ‘The need to develop palliative care education’ *(f = 414);* and ‘Meaning of palliative care and its education’ (*f* = 110) (Table 4).

**Table 4:** Unifying category: Development needs and views of palliative care education

Main category	Category	Subcategory
The need to develop palliative care education *(f = 414)*	More palliative care education in general *(f = 270)*	More palliative care education *(f = 175)* More resources to palliative care education *(f = 36)* Palliative care education should be more extensive *(f = 28)* Obligatory course available to all students *(f = 16)* Clear need to develop the education *(f = 11)* Palliative care education should be provided to all students *(f = 4)*
	More comprehensive and coherent education (*f* = 109)	Palliative care education as an own course (*f* = 67) Palliative care should include deep learning (*f* = 15) Comprehensive education of all aspects of palliative care (*f* = 15) More possibilities to complete elective studies (*f* = 7) Diverse teaching of palliative care (*f* = 5)
	Integrate palliative care clinical practice into the studies (*f* = 35)	Palliative care integrated into clinical practice (*f* = 20) Clinical practice in palliative care settings (*f* = 8) Possibility to care for palliative care patients (*f* = 7)
Meaning of palliative care and its education *(f = 110)*	Importance of palliative care education *(f = 55)*	Palliative care is an important topic *(f = 40)* Palliative care education should be an essential part of nursing education *(f = 5)* Palliative care should be one of the most important topics in education *(f = 5)* Palliative care is a broad topic *(f = 5)*
	The meaning of palliative care *(f = 33)*	Palliative care will be required regardless of the workplace *(f = 12)* Palliative care affects different patient groups *(f = 6)* Just one chance to succeed *(f = 5)* Palliative care is a multidimensional issue *(f = 4)* Palliative care deserves attention *(f = 3)* Palliative care is a valuable type of care *(f = 3)*
	The importance of palliative care in the nursing profession *(f = 22)*	Palliative care is a pivotal part of nursing (*f* = 11) Every nurse should have basic competences in palliative care (*f* = 9) Palliative care competences build professional growth (*f* = 2)

f, number of codes (reduced expressions) included in the categories

The main category ‘The need to develop palliative care education’ (*f* = 414) included three categories and 14 *subcategories*. In this main category, most codes fell under the category ‘More palliative care education in general’ (*f* = 270), which included six subcategories. The subcategories which included the most codes were ‘*More palliative care education’ (f = 175)* and *‘More resources to palliative care education’ (f = 36).* The students also felt that equal access to palliative care education, i.e., ‘*Obligatory course available to all students’* (*f* = 16), was an important area of development. Some examples of the original data are:

*“Definitely more education about this topic into the nursing degree!” 414.*

*“More time resources should be given to palliative care teaching.” 1136.*

*“A separate obligatory own course to all (students) about palliative nursing.” 312*

The main category ‘Meaning of palliative care and its education’ (*f* = 110) consisted of three categories and 13 subcategories. The category that included the most codes was ‘Importance of palliative care education’ (*f* = 55), which included four subcategories, while the subcategory with the most codes was ‘*palliative care is an important topic’ (f = 40)*. Additional subcategories that included numerous codes were: *‘palliative care education should be an essential part of nursing education’ (f = 5);* and *‘palliative care should be one of the most important topics in education’ (f = 5).* The students expressed the following:

*“In my opinion palliative care is a very important topic for nurses.” 739*

*“Palliative nursing is an essential part of education, and it is important to gain knowledge about it.” 874*

*“This subject should be one of the most important to learn about, so that you can feel safe and know what to do when you are in the situation.” w13*

### Preferred types of palliative care education

This unifying category included three main categories, namely, ‘Teaching contents in palliative care education *(f=905),* ‘Teaching methods for learning palliative care’ (*f* = 393), and ‘Placement of palliative care studies (*f* = 81), see Table 5.

**Table 5:** Unifying category: Preferred types of palliative care education

Main category	Category	Subcategory
Teaching contents in palliative care education (*f* = 905)	Encounters in palliative care *(f = 162)*	Guidance to encounter the closest ones *(f = 72)* Guidance to encounter the patients *(f = 59)* Theory and practice of palliative care encounters *(f = 16)* Importance of leisurely and empathic presence *(f = 10)* Importance of genuine encounters *(f = 5)*
	Support in palliative care *(f = 123)*	Knowledge of supporting the closest ones *(f = 42)* More about psychosocial support *(f = 34)* Knowledge of support for patients *(f = 23)* Knowledge of the instrumental support for the patient and family *(f = 7)* Knowledge of patient counselling (*f* = 7) Maintaining hope *(f = 7)* More about supporting the closest ones to participate in care *(f = 3)*
	Holistic pain management *(f = 94)*	More education of pain management *(f = 35)* Education of non-pharmacological pain treatment *(f = 19)* Thorough knowledge of pharmacological pain management *(f = 13)* Knowledge of using patient-controlled analgesia device *(f = 9)* Guidelines to pain management *(f = 8)* Knowledge of pain assessment in palliative care *(f = 6)* Knowledge of the holistic nature of pain *(f = 4)*
	Communication and interaction in palliative care *(f = 73)*	More about interacting with patient and the closest ones *(f = 25)* How to discuss when there are no right words *(f = 9)* How to discuss bad news *(f = 9)* How to discuss with the patient *(f = 8)* How to discuss meaningful issues *(f = 7)* How to discuss with the closest ones *(f = 6)* How to communicate about death with the patient and closest ones *(f = 5)* Practical guidance for interactions *(f = 4)*
	Cultural issues in palliative care *(f = 61)*	Knowledge about multiculturality in palliative care *(f = 34)* Knowledge about multicultural nursing in palliative care *(f = 9)* Knowledge about cultural differences towards death and dying *(f = 8)* Knowledge of the customs of other cultures *(f = 7)* Knowledge of encounters with people from other cultures *(f = 3)*
	The basics of palliative care *(f = 56)*	Clarify the main concepts of palliative and end-of-life care (f = 17) Education about the philosophy of palliative care *(f = 15)* Main contents of palliative care provision *(f = 12)* Identifying the need for palliative care *(f = 6)* Education of basic nursing care as a part of palliative nursing *(f = 6)*
	Special principles of pharmacology in palliative care *(f = 54)*	More knowledge about pharmacology in palliative care *(f = 40)* Knowledge of the special features of pharmacology in palliative care *(f = 8)* Knowledge of the effects and administration of medicine *(f = 6)*
	Advanced care planning, decision-making in palliative care *(f = 48)*	Clarify the concepts of withholding therapies *(f = 19)* More about setting goals of care *(f = 15)* End-of-life decision- making *(f = 12)* ‘Do not resuscitate’ directives *(f = 2)*
	Education of end-of-life care *(f = 31)*	Knowledge about caring for the dying patient and their closest ones *(f = 19)* Caring for the patient and their closest one after death *(f = 8)* Knowledge about palliative sedation *(f = 2)* Knowledge about the symptoms of impending death *(f = 2)*
	Ethical and legal issues in palliative care *(f = 31)*	Knowledge of ethical questions *(f = 24)* Knowledge of values *(f = 4)* Education of legal issues *(f = 2)* Knowledge of ethics in euthanasia *(f = 1)*
	Somatic symptom management in palliative care *(f = 30)*	Care for somatic symptoms *(f = 10)* Assessment in symptom care *(f = 6)* Knowledge of somatic symptoms *(f = 5)* Caring for nausea *(f = 5)* Caring for wounds *(f = 2)* Caring for shortness of breath *(f = 2)*
	Existential issues in palliative care *(f = 26)*	Knowledge about spiritual support *(f = 10)* Knowledge of the meaning of life and existential questions *(f = 10)* Knowledge about different religious views towards death and dying *(f = 6)*
	Self-awareness in palliative care *(f = 25)*	Facing own feelings of death *(f = 8)* Reflection of own feelings regarding care *(f = 7)* Guidance for coping at work *(f = 6)* Guidance on how to cope with difficult situations *(f = 4)*
	Palliative care to different patient groups *(f = 23)*	Children’s palliative care *(f = 10)* Adolescent’s palliative care *(f = 7)* Palliative care in different diseases *(f = 4)* Adult’s and elderly people’s palliative care *(f = 2)*
	Psychological symptom management in palliative care *(f = 21)*	Knowledge of psychological symptoms *(f = 13)* Care for psychological symptoms *(f = 8)*
	Non-pharmacological care in palliative care *(f = 20)*	Overall knowledge about non-pharmacological care *(f = 14)* Different non-pharmacological methods *(f = 6)*
	Palliative care in different settings *(f = 17)*	The care pathway and actors in palliative care *(f = 7)* Providing palliative care at the patient’s home *(f = 5)* Providing palliative care in non-specialized units *(f = 5)*
	Multidisciplinary teamwork *(f = 10)*	Knowledge of multidisciplinary collaboration *(f = 7)* Knowledge of multidisciplinary care *(f = 3)*
Teaching methods for learning palliative care (*f* = 393)	Patient cases and collaboration with working field in teaching *(f = 146)*	Using concrete examples from practice (f = 56) Lectures provided by experts in the field (f = 31) Visits to hospice or palliative care wards (*f* = 26) Using patient cases in education (*f* = 19) Lectures from expert nurses in the field (*f* = 14)
	Multidimensional teaching methods (*f* = 88)	Face-to-face education *(f = 63)* More reflection tasks about the issue *(f = 7)* Online videos about palliative care *(f = 6)* Using e-learning to create flexibility *(f = 5)* Evidence-based education *(f = 5)* Taking into account different learning styles (*f* = 2)
	Experiences and exposure-based teaching (*f* = 49)	Experts by experience telling their story (*f* = 28) Sharing care experiences with the classes (*f* = 10) Teachers sharing their own experiences of palliative care (*f* = 7) Students sharing their own experiences of palliative care (*f* = 4)
	Learning from discussions about palliative care *(f = 46)*	Teacher facilitated discussion about palliative care issues *(f = 31)* Teacher facilitated group discussions *(f = 12)* Teacher facilitated discussions of care encounters and feelings *(f = 3)*
	Skills labs and simulation pedagogy in palliative care education *(f = 40)*	Simulation sessions *(f = 19)* Skills practice through workshops *(f = 12)* Skills training sessions at school *(f = 9)*
	Multidisciplinary teaching and learning *(f = 24)*	Lectures provided by physicians *(f = 8)* Learning together with students from other disciplines *(f = 7)* Lectures provided by chaplains *(f = 6)* Teaching provided by a multidisciplinary palliative care team *(f = 3)*
Placement of palliative care studies (*f* = 81)	Integrated and unifying palliative care education in the curriculum *(f = 35)*	Repeated teaching at different phases of education *(f = 12)* Education as an own entirety *(f = 8)* Palliative care education as a natural part of all education *(f = 6)* Palliative care integrated in different courses *(f = 6)* Teaching after clinical practice *(f = 3)*
	Preparatory teaching from the first semesters *(f = 29)*	Education launched during the first semesters *(f = 16)* Education before the first patient contacts *(f = 8)* Education from the beginning of the studies *(f = 5)*
	In-depth learning during the final semesters *(f = 17)*	Palliative care education integrated into advanced studies *(f = 7)* Palliative care education integrated into the last semesters of studies *(f = 7)* Cases and simulations integrated into advanced studies *(f = 3)*

f, number of codes (reduced expressions) included in the categories

The main category ‘Teaching contents in palliative care education’ (*f* = 905) included 18 categories and 78 subcategories. The category with most codes was ‘Encounters in palliative care’ (*f* = 162), which consisted of five subcategories, with the subcategories ‘*guidance to encounter the closest ones’ (f = 72)* and *‘guidance to encounter the patients’ (f = 59)* including the most codes. The students also highlighted the need for more ‘*theory and practice of palliative care encounters’ (f = 16)*. The students shared the following:

*“Education of how we as nurses can encounter… the closest ones.” 1135*

*“More education of encounters with dying patients.” 561*

*“It would be good to get more theory and advice concerning humane encounters.” 101*

The main category ‘Teaching methods for learning palliative care’ (*f* = 393) consisted of six categories and 25 subcategories. The category that included the most codes was ‘Patient cases and collaboration with working field in teaching’ (*f* = 146), and it included five subcategories. The three subcategories with the most codes demonstrated which aspects of teaching the students preferred, namely, teachers ‘*using concrete examples from practice’ (f = 56), lectures provided by experts in the field (f = 31),* and ‘*visits to hospice or palliative care wards’ (f = 26).* As examples, the students stated the following:

*“To orientate with palliative care, concrete examples from the working environment should be used so that it could be concretely understood.” 308*

*“An experienced end-of-life / palliative care professional could come and talk about the topic.” 684*

*“… by visiting hospice and palliative care units during the education.” 198*

The main category ‘Placement of palliative care studies’ (*f* = 81) consisted of three categories and 11 subcategories. The category which included the most codes was ‘Integrated and unifying palliative care education in the curriculum’ (*f* = 35), which included five subcategories. The subcategories with the most codes highlighted various aspects of palliative care learning, namely, ‘*repeated teaching at different phases of education’(f* = *12)*, ‘*education as an own entirety’ (f = 8)*, and *‘palliative care education* should be *a natural part of all education’ (f = 6),* which highlighted how palliative care could be integrated into undergraduate nursing studies.

*“The topic could be repeated during the nursing studies.” 282.*

*“It should also be one own teaching entity, where discussion about the topic will be made.” 337*

*“It could be a small part of every teaching session, making it “naturally” applicable to working life.” 618*

### Factors that promote or hinder palliative care learning

This unifying category included two main categories: ‘Factors that facilitate palliative care learning’ (*f* = 66); and ‘Barriers to palliative care learning’ (*f* = 335) (Table 6).

**Table 6:** Unifying category: Factors that promote or hinder palliative care learning

Main category	Category	Subcategory
Factors that facilitate palliative care learning (*f* = 66)	Previous clinical experience about palliative care *(f = 31)*	Palliative care clinical practice *(f = 15)* Work experience from clinical settings *(f = 14)* Mentoring in clinical practice *(f = 2)*
	Obtained formal education *(f = 25)*	Elective studies regarding palliative care *(f = 10)* Education obtained while studying for a former health care degree *(f = 10)* The expertise of the teacher *(f = 5)*
	Intrinsic motivation to learn about palliative care *(f = 10)*	Personal interest in palliative care *(f = 5)* Thesis completed on the subject of palliative care *(f = 4)* Personal experience of palliative care *(f = 1)*
Barriers to palliative care learning (f = 335)	Insufficient amount of education *(f = 119)*	Too little education of palliative care *(f = 92)* Too superficial education *(f = 14)* No education of palliative care *(f = 7)* Too concise course of palliative care *(f = 6)*
	Insecurity about own performance in palliative care *(f = 56)*	Too little competence to provide palliative care *(f = 21)* Hard to encounter the dying patients and the closest ones *(f = 8)* Everyone don’t have enough interaction skills to face the dying person *(f = 7)* Unpreparedness how to perform in difficult situations *(f = 6)* Palliative care can be frightening (f = 5) The topic is difficult *(f = 5)* Difficult to face death *(f = 4)*
	Discrepancy between teaching methods *(f = 43)*	Too much online learning *(f = 18)* Too much self-learning *(f = 17)* Too much group work *(f = 6)* Classes are too long for such a serious topic *(f = 2)*
	Insufficient structure of education *(f = 37)*	Fragmented entities do not form an overall picture *(f = 16)* Death is hidden from the nursing curriculum *(f = 10)* The teaching was carried out too fast *(f = 8)* No education obtained because of school change *(f = 3)*
	Shortcomings of competences and clinical learning (*f* = 35)	Deficiency in provision of palliative care in the working field *(f = 11)* Lack of palliative care competences among nursing staff *(f = 10)* Have not faced or cared for palliative care patients *(f = 7)* The work environment is responsible for too much of the learning *(f = 4)* It is difficult to face patients due to lack of prior knowledge *(f = 3)*
	Impractical content of the education (*f = 27)*	Education does not develop the competences needed in work life *(f = 7)* Outdated educational contents *(f = 6)* The educational content concentrates too much on the dying phase *(f = 5)* Deficiencies in the contents *(f = 5)* Insufficient teaching on the care of different diseases and symptoms *(f = 4)*
	Teacher’s insufficient competences on the subject *(f = 18)*	Teachers lack sufficient competences *(f = 10)* Teaching is deficient *(f = 8)*

f, number of codes (reduced expressions) included in the categories

The main category ‘Factors facilitating palliative care learning’ included three categories and nine *subcategories*. The category with the most codes was ‘Previous clinical experience about palliative care’ (*f* = 31)*;* under this category the subcategory ‘*palliative care clinical practice’ (f = 15)* included the most codes. The students also expressed that ‘*work experience from clinical settings*’ *(f = 14)* and the possibility to achieve ‘*mentoring in clinical practice (f = 2)* were conducive to palliative care learning. The students expressed:

*“I have completed my clinical practice in a hospice, and it was an eye-opening learning experience.” w41*

*“Palliative care and end-of-life care have become familiar to me when working as a nurse during my studies.” w32*

*“Good instructors in working life and clinical practice taught me a lot.” 1113*

The main category ‘Barriers to palliative care learning’ included seven categories and 31 subcategories. The category that included the most codes was ‘Insufficient amount of education’ *(f = 119),* which included four subcategories. The following subcategories included the most codes: ‘*too little education of palliative care’ (f = 92);* ‘*too superficial education’ (f = 14) and ‘ no education of palliative care’ (n = 7)* Some original expressions under:

*“There is too little palliative care education during the studies.”* 48

*“The teaching of palliative nursing was too superficial.”302*

*“ … I did not receive any education of palliative care” 68*

## Discussion

The participating students expressed their perspectives of both the current state of palliative care education and the development needs for this aspect of nursing education. The students often shared that undergraduate nursing studies should generally include more palliative care education. These perspectives can reflect to the variation of the palliative care education in the Finnish UASs [21]. It is noteworthy that the students regarded palliative care education as an important and essential topic which should be integrated into the nursing curriculum. As palliative care should be incorporated in the early stages of caring for a person with a chronic illness based on the need not on the diagnosis, prognosis or settings of care, it is most likely that nurses encounter palliative care patients during their career [5]. Therefore, it is important that all students would be prepared for the care when graduating. The students highlighted the importance of practical aspects of palliative care, i.e., knowledge of how to manage encounters with patients, which can be gained by meeting real patients and being taught by experts from the field such as specialist nurses and physicians. In general, the education delivered by experts from the field should be better utilized, since this probably differs between the UASs in Finland.

The students also expressed their views of which types of palliative care education they preferred. Notably, the students described various aspects of palliative care that they hoped should be integrated into nursing education. This may reflect the wide range of competences nurses need to provide high-quality palliative care. The palliative care aspects mentioned by students agree with earlier descriptions of palliative care competences [19, 43–47]. In addition to the content of palliative care education, the students specified which teaching methods they preferred. Earlier literature has confirmed that various methods are effective at enhancing students’ palliative care learning [22, 23, 48], with this study providing a unique, detailed view of which aspects of palliative care and which teaching methods they prefer.

The undergraduate nursing students also shared their views of which factors promote and hinder their palliative care learning. They expressed that clinical practice in palliative care settings and mentoring by experienced nurses facilitate the learning process. A supportive mentor-student relationship, including guidance and role-modelling, has been identified to facilitate students’ clinical learning [49]*,* while other research has stated that staff support is crucial to good learning experiences [17, 50]. First-hand experience in caring for dying persons was found to be associated with positive attitudes towards end-of-life care [51]. For students to gain positive learning experiences about palliative care, they should be afforded more opportunities to attend clinical practice placements and receive instruction and support from experts in the field. This is essential, since students also expressed that staff members with insufficient palliative care competences can hinder learning.

Another subject which was reported to be both a facilitator and barrier to learning was the teacher’s competence, i.e., a competent teacher facilitated the learning process while incompetent teachers served as a barrier to learning. Student expressed that a competent teacher has due-to-date knowledge of palliative care and experience of the subject to share concrete examples to the students. Competent teachers are also wished to have an open and supporting attitude to the students’ feelings and concerns of the subject. The importance of a competent educator has been highlighted in a European guide for the development of palliative nurse education [19]*.* Furthermore, previous studies have also stated that teachers need additional palliative care education [52] and that the lack of competent teachers can significantly hinder high-quality education [53]. Hence, educational institutions should assess and ensure the competence of teaching staff, as this is related to the quality of undergraduate nursing education.

The students also identified feelings of unpreparedness and fear of the topic as barriers to learning. Unpreparedness, feelings of vulnerability and fear have also been reported in earlier research [24, 54, 55]*.* The expression of uncertainty and feelings of unpreparedness can often be explained by insufficient experience and the lack of education [17, 56–58]*.* The barriers to learning reported by the undergraduate nursing students participating in this study revealed certain areas of palliative care education which must be improved in nursing curricula.

Some future implications for research were identified during the study. This study concerned final-year students, who may have limited understanding of all needs of the education to prepare them to the future work with palliative care patients. Therefore, as a future implication for research it would be important to focus also on graduated nurses. This study gave a comprehensive summary of the phenomenon, hence, to get a more in-depth view of the phenomenon face-to face or focus-group interviews would further bridge the gaps in the knowledge of the palliative nursing education. In addition, relatively little is known about teachers’ competence and requirements to provide quality palliative care education. Even though this was identified as a facilitating or hindering factor in learning palliative care by the students, more focus on this should be put in future research.

This study gave a comprehensive summary of nursing students views of palliative care education development needs. The research also provides information on what factors should be changed or strengthen to improve students learning. The results can be utilized when developing the undergraduate nursing curriculum. This study gives information of the preferred placement of the education, namely thorough the nursing education. In addition, it provides an overview of students’ perceptions of the preferred teaching contents and methods. It calls to increase the amount of palliative care education and for more research of the phenomenon, as well.

### Strengths and limitations

Several aspects strengthened the trustworthiness of this study. The chosen method was suitable for the purpose of this study*.* Moreover, data saturation was achieved during the analysis [38]. The credibility was strengthened by reporting in detail the sampling, data collection and analysis process. The clarity of the open-ended question was pretested, and the sample included final-year nursing students from all UASs across Finland. Therefore, it can be assumed that the study population had experiences of the achieved palliative care education in nursing studies. Furthermore, dependability was strengthened by presenting an example of the analysis process (Table 3) and tables and figures of categories identified through content analysis (Tables 4, 5 and 6, Fig. 1). It should also be noted that the researchers constantly discussed the analysis and findings throughout the study. Confirmability was strengthened by focusing on the manifest content so that the results would represent the views of the students [37]. The participants represented students from all UASs and the data was rich which made it possible to make a comprehensive summary of the phenomenon, which strengthen the transferability of the results. In addition, the sampling and inclusion criteria are described carefully in the manuscript. The authenticity of the results was strengthened by providing authentic citations from the collected data [30, 59]*.*

The research also included certain limitations which could weaken the trustworthiness of the study. For instance, the questionnaire was answered anonymously, and answering the open-ended question was completely voluntary. Hence, it is impossible to know the reason(s) why certain students refused to answer this question. Furthermore, there was no possibility to ask any further questions from the students or return the findings to the students for comments or corrections [30]. In addition, because the students were not yet graduated, they may not possess all the understanding of what their future competence needs would be when caring for patients in palliative care, which can lead to a limited vision of the education needs. It should also be noted that several subcategories consisted of a small number of codes; however, they are important because they show different aspects of the studied phenomenon.

## Conclusions

This study provides evidence that palliative care education in undergraduate nursing curricula still needs to be developed in terms of the amount, content, methods and integration into the programs. The students’ responses revealed feelings of unpreparedness to provide palliative care even though the nursing students highlighted the importance of palliative care as a topic in their education. Students should have enhanced access to clinical placements or visits to palliative care units to facilitate their learning of palliative care. Furthermore, a teacher’s competence is also linked to the students’ learning processes. This study gives detailed information about nursing students’ perspectives on palliative care education. Based on the results of this study, we suggest that final-year nursing students should gain valuable insight into different aspects of palliative care through their nursing education, and this can be ensured by using effective teaching methods. Therefore, the presented results are of great value to professionals and decision-makers who are planning to integrate palliative care into the undergraduate nursing curriculum.

## Supplementary Information

**Additional file 1.** COREQ- checklist.

## Data Availability

The original data of the current study are not publicly available due to the terms of the achieved research permits from the UASs and to ensure the study participants that the data will be retained confidential. Within the limits of confidentiality, more detailed, but anonymous, data is available from the corresponding author on request.

## Abbreviation

UAS: University of Applied Sciences
